# In vitro safety assessment of alkyl lactate esters in human umbilical vein endothelial cells (HUVECs)

**DOI:** 10.1016/j.toxrep.2022.11.008

**Published:** 2022-12-05

**Authors:** Fatemeh Javaheri-Ghezeldizaj, Maryam Ghaffari, Jafar Ezzati Nazhad Dolatabadi, Parvin Dehghan

**Affiliations:** a Student Research committee, Department of Food Science and Technology, Faculty of Nutrition and Food Sciences, Nutrition Research Center, Tabriz University of Medical Sciences, Tabriz, Iran; b Pharmaceutical Analysis Research Center, Tabriz University of Medical Sciences, Tabriz, Iran; c Drug Applied Research Center, Tabriz University of Medical Sciences, Tabriz, Iran; d Nutrition Research Center, Faculty of Nutrition and Food Sciences, Tabriz University of Medical Sciences, Tabriz, Iran

**Keywords:** CL, Calcium lactate, DAPI, ECs, 4′6-diamidino- 2-phenylindoleEndothelial cells, HUVECs, Human umbilical vein endothelial cells, MTT, Thiazole Blue Tetrazolium Bromide, SL, Sodium lactate, Food additive, Cytotoxicity, Genotoxicity, HUVECs, Apoptosis

## Abstract

Safety assessment requires information on both the chronic and acute effects of chemicals. Chemical materials such as food additives have become one of the most critical and compelling issues in the continuing debate on food safety. Calcium lactate (CL) and Sodium lactate (SL) are approved chemicals used in various products. The present study, in vitro study, was designed to evaluate the cyto-genotoxicity effects of CL and SL on human umbilical vein endothelial cells (HUVECs). The cytotoxicity effect of these additives was determined by MTT and Annexin V-FITC apoptosis assays. Besides, genotoxicity parameters such as morphological change of DNA and DNA fragmentation were studied with DAPI staining and DNA ladder assays, respectively. The results showed that the growth of HUVECs decreased upon treatment with CL at higher concentrations, but SL did not significantly alter HUVECs cell number. Annexin V-FITC apoptosis assays revealed that CL could induce cell death based on necrosis rather than apoptosis. In SL, the early and late apoptosis was not considerable in treated cells. Exposure to CL and SL did not lead to morphological change and DNA fragmentation in the HUVECs cell line. Overall, it is concluded from these results that CL and SL have not been cytotoxic and genotoxic effects in low concentrations in human umbilical vein endothelial cells in vitro.

## Highlights

• Cyto-genotoxic effects of CL and SL were investigated in vitro.
• This is the first study examining their cyto-genotoxic effects in human umbilical vein endothelial cell.
• Proliferation of HUVECs treated with CL and SL decreased in a time and dose-dependent manner especially at high concentrations.
• CL and SL did not induce morphological change and DNA fragmentation in the DAPI staining and DNA ladder assays.

## 1 Introduction

**Fig. 1:**
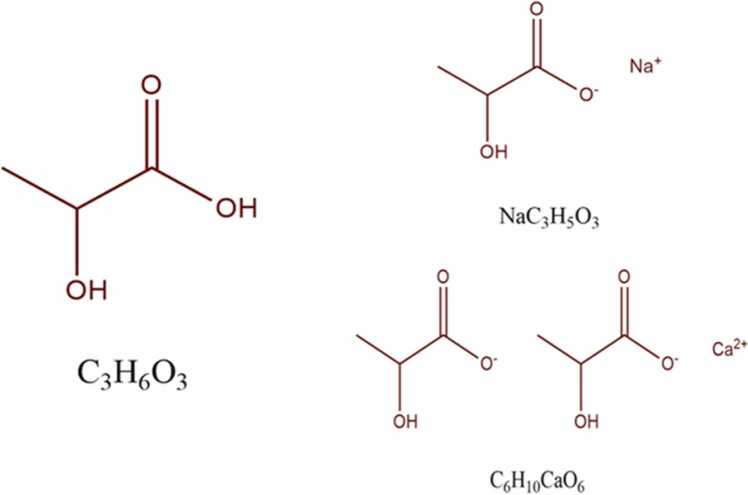
Molecular structure of lactic acid and salts.

Lactic acid and salts (Fig. 1) such as SL and CL are regarded as a food-grade preservative, which is generally recognized as safe (GRAS) by the Food and Drug Administration (FDA). As a result, these additives are widely used in various products, including foodstuffs, cosmetics, and pharmaceutics [17], [25]. CL, known as calcium-L-2-hydroxy-propionate, is produced synthetically by the reaction of purified L-Lactic acid and lime. This additive has been authorized as a preservative (Fig. 1; E 327) with antioxidant, antimicrobial, and stabilizing properties, and also a firming agent, acidity regulator, and flavour enhancer in the process of a wide range of foodstuffs such as potato, vegetable snacks and sweetened crackers [25], [30].

SL (2-Hydroxy propionic acid sodium salt; E325) was approved as a direct food ingredient with antimicrobial, antioxidant, emulsifier, humectant, acidifying agent, and flavour enhancer properties at a 2–3% recommend level [18]. It is worth noting that "Not limited" acceptable daily intake (ADI) was defined for lactic acid, CL, and SL by JECFA (Joint FAO/WHO Expert Committee on Food Additives) [25]. In recent years, the cyto-genotoxic effect of synthetic food additives on public health has been well documented in the literature. Such contradictory information gives rise to doubt about the safety of food additives [2]. Besides, consumers' concerns increased about the potential risks of synthetic food additives by developing awareness of the risks of using these materials [31]. Hence, a safety assessment of food additives is carried out to get authorization for adding them to food. In the field of food additive assessment, the European Food Safety Authority (EFSA) has made a re-evaluation program under regulation (EC) 257/2010 to represent safety evaluation of all food additives which have been authorized and used in foodstuffs [4]. In 1978, the FDA concluded that increased lactate intake from processed foods might potentially raise the adverse effect on human populations in the future (Panel [6]). The Joint FAO/WHO Expert Committee on Food Additives (JECFA) conducted the last risk assessment of lactic acid and CL as feed additives in 2019, which resulted in no conclusions on poultry safety and for pre-ruminants (Panel [7]). According to the literature review, most of these studies were old. On the other hand, with the advent of modern approaches to toxicology, in vitro studies have an essential role in evaluating the chronic and acute effects of chemicals [15].

Endothelial cells (ECs) are the frontrunners in the blood lumen to confront the toxicants. This critical component is still missing and needs urgent attention to understand the toxicity of the chemicals in ECs. HUVECs are among one the most used in vitro models for ECs. The primary isolated HUVECs are probably the most popular ECs used in research because human umbilical veins are relatively more available than other blood vessels [24], [3]. In vitro, cyto-genotoxicity tests aim to determine whether a compound, product or environmental factor induces damage in the genetic material of cells or organisms. To the best of our knowledge, the cyto-genotoxic results of CL and SL have not been reported on HUVECs cells. Consequently, to plug the data gap, we aimed to expose HUVECs with sodium lactate and calcium lactate to investigate the (i) proliferation inhibition by MTT assay and flow-cytometer assays, (ii) DNA damage by DNA ladder, and (iii) chromatin damage by DAPI staining.

## 2 Materials and methods

### 2.1 Chemical

HUVECs were supplied by the Pasteur Institute-national cell bank of Iran. CL and SL were obtained from Sigma-Aldrich (purity ∼98%). The SPL Life Sciences Co (Gyeonggi-do, Korea) provided flasks and cell culture plates. The Apoptosis detection kit AnnexinV-fluorescein isothiocyanate (FITC) was purchased from EXBIO Praha, a.s. (Czech). Dulbecco’s Modified Eagle Medium (DMEM), penicillin-streptomycin (catalogue number: 10566016), DAPI (4′6-diamidino- 2-phenylindole), EDTA (Trypsin-ethylene diamine tetra acetic acid), FBS (fetal bovine serum), PBS (phosphate buffered saline, pH 7.2), MTT (Thiazole Blue Tetrazolium Bromide), dimethyl sulfoxide (DMSO) and other chemicals were purchased from Sigma Chemical Corp., Germany.

### 2.2 Cell culture

HUVECs were cultured in a complete medium containing DMEM (high glucose), 10% FBS and 1% penicillin (100 U/ml)/streptomycin (100 μg/ml). The cells were maintained at 37 ^◦^C in a relative humidity of 95% and 5% CO_2_ concentration. Cells were passaged every 3–4 days (by seeding at 1:3 ratios) for a maximum of 8 times. After reaching a confluency of 80%, the cells were passaged by 2 ml trypsin enzyme and seeded to 6-well plates with 3 × 10^5^ cells per well or 96-well plates at a density of 15 × 10^3^ cells per well. Each well contained 200 μl of cell suspension, and the plates were incubated for 24 h at 37 °C under 5% CO_2_ to obtain a monolayer culture. After a 24 h incubation period, the old medium was replaced by a fresh medium. The CL and SL stock solutions were prepared at a concentration of (100 mM) by dissolving an appropriate amount of them in doubly distilled water and then filtered before adding them to HUVECs.

### 2.3 MTT assay

The MTT assay measure viability, cell membrane integrity, and cell proliferation. The reduction of the tetrazolium salt, 3-(4,5-dimethylthiazol-2-yl)− 2,5-diphenyltetrazolium bromide (MTT) to a blue formazan crystal is facilitated by mitochondrial dehydrogenase and NADPH-dependent cellular oxidoreductase enzymes [1]. First, cells were seeded at a density of 15 × 10^3^ and incubated for a day before exposure to CL and SL. Then, different concentrations of CL and SL ranging from 115 μM to 16,000 μM were prepared and added to each well and then incubated for 24 h and 48 h at 37 °C. Next, the prepared solution of MTT in PBS (0.5 mg/ml) was added to each well and re-incubated for another four hours in a humidified incubator. After removing the medium, DMSO (200 μl) was added to dissolve formazan crystals formed by the viable cells. Finally, after 20 min, the spectrophotometric plate reader (BioTek Instruments Inc, Vermont, USA) was used for UV absorbance measurements at 570 nm (test wavelength) and 630 nm (as reference wavelength) using a microplate reader (Synergy 2; BioTek Instruments, Inc., Germany). By the MTT method, cell numbers were obtained as absorbance values. The results were expressed as IC50 values (50% inhibitory concentration). The percentage of cell viability was calculated relative to negative controls, and results were presented as mean Cell Viability (%) ± standard deviation of at least three independent experiments. All experiments were set up in three replicates[11], [27].

### 2.4 Annexin V/PI staining for evaluation of apoptosis and necrosis

FITC-labeled Annexin V assay was carried out to detect apoptosis and necrosis in HUVECs treated with CL and SL. In brief, HUVECs were seeded at a density of 3 × 10^5^/well in a 6-well plate and incubated for 24 h. After reaching a confluency of 70%, cells were exposed to an IC50 concentration of CL and 4,000 μM of SL for 48 h. Then, the cells were washed with PBS three times, followed by trypsinization (1 ml), incubation for 5 min at 37 °C, and resuspended in 100 μl of 1X binding buffer. Next, 10 μl of Annexin V- FITC and 5 μl of PI were added to the cell suspension and then incubated at 25 °C in the dark for 15 min to stain the cells before analysis by flow cytometry. In the final step, 400 μl of binding buffer was added, and the cell samples were quantified using a flow cytometer by measuring at 515–545 nm for FITC (green) and 600 nm for PI (red). Cells were then analyzed by flow cytometry (FACScan; Becton Dickinson Immunocytometry Systems). The percentage of apoptosis and necrosis was estimated using the Graph Pad Prism 9.0 software program [26].

### 2.5 Genotoxicity assays procedure

Genotoxicity of the CL and SL was evaluated by genotoxicity assays, including DNA fragmentation and DAPI staining.

#### 2.5.1 Fluorescent staining of the nucleus by DAPI

DAPI staining is one of the most commonly used fluorescent-based assays to evaluate the effect of treatments on DNA content, and consequently, the corresponding cells [23]. Briefly, HUVECs were seeded at a density of 15 × 10^3^ cells/well in a 96-well plate for 24 h. Following treatment with CL (1,535 μmol/L), SL (4,000 μmol/L), and DMSO (200 ml) as a positive control, the treated cells were fixed with 4% paraformaldehyde for one hour at 25 °C. After washing three times with PBS, fixed cells permeabilized with 0.1% (w/v) Triton X-100 for 5 min and then washed again with PBS. Next, 20 μM of DAPI was used to stain permeabilized cells and then incubated for 30 min. Finally, cells were observed under a fluorescent microscope to image fragmentation and nuclear condensation at the emission of 460 nm and excitation of 360 nm [16].

#### 2.5.2 DNA fragmentation assessment

DNA gel electrophoresis was used to determine the presence of internucleosomal DNA cleavage. HUVECs treated with CL and SL were evaluated for DNA fragments or smears in agarose gel. All stages were performed based on a previously published procedure. In brief, HUVECs were seeded into a 6-well plate at a density of 3 × 105. After incubation overnight, IC50 concentration of CL and 4,000 μM of SL were used to treat HUVECs for 48 h. Next, treated cells were harvested and suspended for 15–30 min in the 250 μl lysis buffer containing 10 mM EDTA, 50 mM Tris base, 5 units RNase, and 0.5% sodium dodecyl sulfate (SDS) at 37 °C and pH 7.4. Then, the chloroform-isoamialcohol stage was applied to denature total proteins with 500 ml, followed by separating total DNA into the upper phase of suspension (centrifugation at 12,000 rpm). Finally, total DNA was precipitated with an equal volume of isopropanol and then electrophoresed in a 2% agarose gel containing 2 ml/100 ml SYBR Safe DNA gel stain (1 h at 70 V/30 mA). The gel was examined and visualized under UV light [16].

### 2.6 Statistical analysis

All results were performed with three independent experiments. Data processing was applied to evaluate significant differences for the measured variables using one-way variance analysis (ANOVA). The statistical significance was defined as a P-value of less than 0.05. Graph Pad Prism 9.0 software was applied to create Figures and calculate IC50 of treated samples.

## 3 Result

### 3.1 Cytotoxicity of MTT assay results

**Fig. 2:**
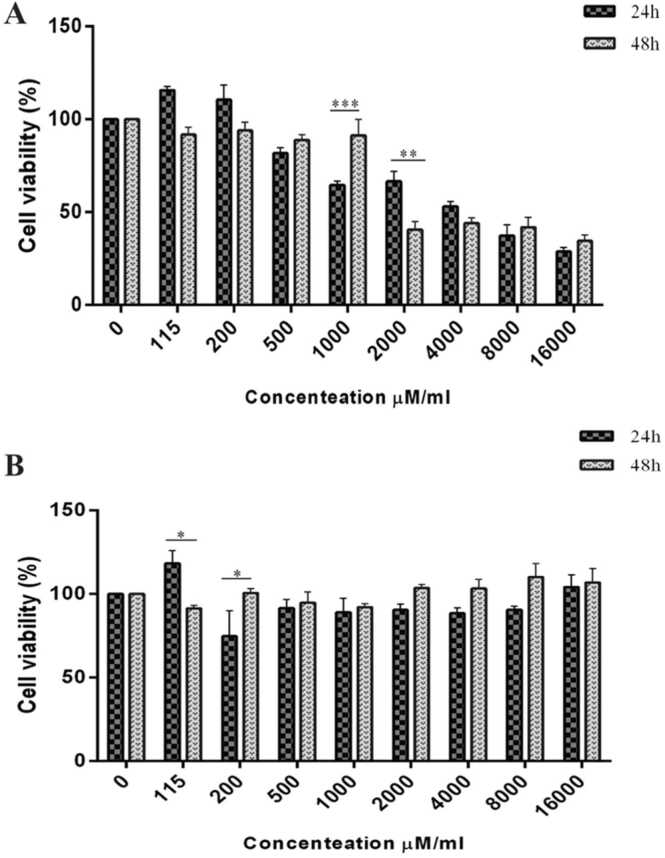
Effect of different concentrations of A) CL and B) SL ranging from 115 to 16,000 µg/ml on cell viability of the HUVECs after 24 and 48 h. The results were shown based on cell numbers related to the control group.

To investigate the possible effects of CL and SL on cell viability using MTT assay, various CL and SL concentrations, ranging from 115 µg/ml to 16,000 µg/ml, were used to treat HUVECs for 24 and 48 h. Based on obtained results in Fig. 2, The MTT assay demonstrated that SL did not significantly alter HUVECs cell number. Also, HUVECs treated with CL exhibited a reduction in growth in a time and dose-dependent manner, which was more evident at high doses compared to the control group. The IC50 of 3,200 μM and 1,535 μM was attained after 24 and 48 h of treatment with CL, respectively, whereas SL could not reduce the proliferation of HUVECs up to 50%. However, we used 4,000 μM SL in experiments instead of IC50 to investigate more effects.

### 3.2 Flow cytometry annexin V/PI measurement

Flow cytometry quantified the percentage of apoptotic cells as a standard technique. In addition, the double-staining with Annexin V-FITC and PI was carried out to discriminate the mechanisms of cell toxicity (apoptosis or necrosis) in the treated and untreated groups. Annexin V is a calcium-dependent phospholipid-binding protein with a high affinity for phosphatidylserine (PS) on the cell surface. Translocation of the PS from the inner side of the plasma membrane to the outer side upon occurring apoptosis triggers cell death. Generally, both Annexin V and PI-negative cells are considered unstained cells (live cells), and necrotic cells are PI-positive and Annexin V negative ones.

**Fig. 3:**
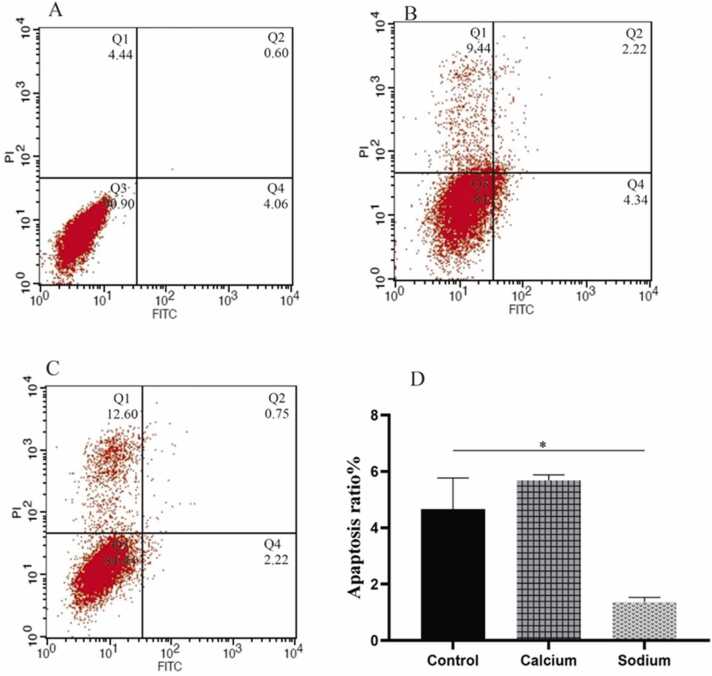
Apoptosis detection of HUVECs in A) the control group B) CL and C) SL after exposure with IC50 concentration after 48 h performed by FITC-labeled Annexin V/PI flow cytometry assay.

Moreover, cells colored with Annexin V and PI are categorized as apoptotic cells, whereas cells in the early stage of apoptosis are PI negative and Annexin V positive [9]. The extent of cell death observed using this method was the same as that measured by the MTT assay. Fig. 3 showed that 9–12% of HUVECs treated with CL and SL led to necrosis. Besides, 6.56% and 2.97% of cells treated with CL and SL experienced apoptosis after 48 h. However, 4.44% of control cells were in stages of necrosis, and 5.2% showed apoptosis after the same treatment period. We observed small necrosis and apoptosis in HUVECs cells treated with CL and SL compared to unstained cells. However, CL caused the lowest percentage of apoptosis ratio compared to the control group. So, CL could induce cell death based on necrosis rather than apoptosis following flow cytometry results. This result can be explained by the fact that the decrease in cell viability can be due to apoptosis by CL.

### 3.3 Fluorescent staining of nuclei by DAPI

**Fig. 4:**
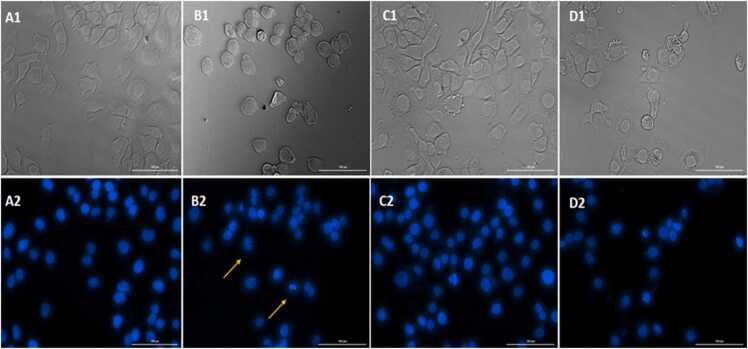
Fluorescent and light microscopy images of HUVECs stained with DAPI. (A1 & A2) Untreated (B1 & B2) Positive control treated by 5% DMSO, (C1 & C2), and (D1 & D2) treated cells with CL and SL, respectively. Arrows showed chromatin and DNA fragmentation in the nucleus of HUVECs.

DAPI is one of the most common assays to evaluate the morphological change of apoptotic cells, binding to A-T-rich regions of double-strand DNA [23]. Generally, DNA cleavage and chromatin fragmentation are signs of apoptosis induction evident in treated cells. In this study, HUVECs were stained with this fluorescent dye to depict changes in nuclear morphology and chromatin distribution upon treatment with IC50 concentration of CL and 4,000 μM of SL. Fig. 4 shows representative fluorescence microscopy images of the DAPI-stained cells after 48 h exposure to 5% DMSO (positive control) and IC50 concentration of CL and 4,000 μM of SL. As demonstrated, CL and SL did not cause DNA damage and fragmentation, chromatin condensation, and fragmentation in the nucleus of treated cells. Therefore, morphological changes of the nucleus in cells treated with CL and SL were the same as in untreated cells. Based on the results from the genotoxicity assessment by the DAPI staining, it can be revealed that there was no apoptosis occurrence upon treatment of the cells with these additive.

### 3.4 DNA ladder assessment

**Fig. 5:**
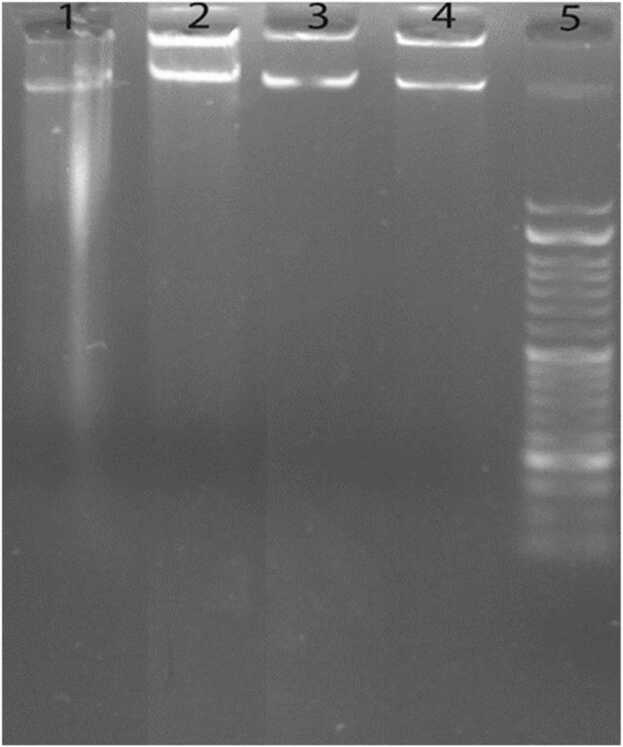
DNA fragmentation in HUVECs performed by DNA ladder assay. Lane 1 and 2 are the negative and positive control groups. CL and SL are Lane 3 and Lane 4, respectively. Lane 5 is a DNA marker.

Finally, to further confirm the results, we examined DNA fragmentation assay as the most dependable method for distinguishing apoptotic cell death from necrotic one in HUVECs. Therefore, we studied the DNA fragment patterns and DNA ladder formation using DNA ladder pattern widening from 180 to 200 bp on agarose gel electrophoresis. The obtained results in Fig. 5 demonstrate that IC50 concentration of CL and SL in the concentration of 4,000 μM did not cause any fracturing in DNA structure or smear compared to the control group. These results confirmed the findings of the DAPI staining assay. The formation of the DNA ladder illustrates the occurrence of DNA breakage and fragmentation. Given DNA ladder results, treated cells with CL and SL did not break, condensed, and remodeled DNA structures. Also, it can be demonstrated that cell growth decreased upon treatment with these additives, which was not associated with cell apoptosis.

## 4 Discussion

The food industry is one of the fastest-growing economic sectors in the world, generating high competitiveness among producers in search of meeting new consumer demands. For that, they try to produce attractive foods from a hygienic, nutritional and sensory point of view [10,29]. However, aiming at becoming competitive, they increasingly use food additives, which are any ingredient intentionally added to foods to modify their physical, chemical, biological or sensory characteristics without nutritional purpose. Pressman et al., ($year$) [22]. One of the foremost traits of synthetic food additives is their possible side effects in vivo. Several studies have shown that some food additives may have toxic, mutagenic, and genotoxic effects. The leading cause of toxicity of these types of materials is related to the imbalance in the cell’s physiology and signalling. Consequently, apoptosis, as one of the possible ways to remove defective cells, can result. Therefore, accurately measuring cytotoxicity and genotoxicity is a precious tool in identifying compounds that might pose certain health risks in humans [11]. This study investigated the possible cyto-genotoxic effect of CL and SL using MTT, DNA ladder, DAPI staining, and flow cytometry in HUVECs. The results obtained in this investigation showed that CL could lead to cell growth inhibition dose and time-dependently. In line with our results, Eskansani et al., in 2014 and Karimi et al., in 2019, assessed the cytotoxic effects of tert-butyl hydroquinone (TBHQ) on HUVECs. They found that treatment with TBHQ reduced the proliferation of studied cells time and dose-dependently [12], [8]. In a study carried out by Mohammadzadeh-Aghdash in 2018, it was shown that sodium acetate, sodium diacetate, and potassium sorbate decreased the growth of HUVECs compared to untreated cells [19]. Similarly, Hamishehkar et al., using an in vitro assay with HUVECs, showed that propyl gallate has cytotoxic effects at concentrations of 1 × 10^3^ in 24 h [11]. It is essential to discriminate between necrosis and apoptosis to provide an adequate description of the selective toxicity of chemical compounds [14]. Flow cytometry apoptosis assays revealed that CL could induce cell death based on necrosis rather than apoptosis. In SL, the early and late apoptosis was not considerable in treated cells. The Genotoxicity of chemicals such as food additives is considered as one of the most severe side effects on human beings. If a chemical reacts with nuclear DNA, it may be carcinogenic and mutagenic to the exposed organism [5]. Cells encountering apoptosis show a reduction of cytosolic volume, chromatin condensation, and a typical ladder-like electrophoretic DNA appearance due to DNA fragmentation into nucleosome-length fragments of 180–200 bp and multiples thereof [28]. Herein, we examined DNA fragmentation as a possible mechanism for the induction of cytotoxicity in cultured HUVECs. The DNA electrophoretic mobility experiments showed that IC50 concentration of CL and 4,000 μM of SL did not cause any fracturing in DNA structure or smear compared to the control group. DAPI is a standard nuclear stain which binds strongly to the DNA double helix at adenine thymine rich chromosomal regions. It is used to study and quantify DNA in cellular systems as staining nucleic acids [13]. Therefore, the nuclear morphology changes, including chromatin condensation and fragmentation of HUVECs due to the treatment with CL and SL, were investigated using DAPI staining. Upon microscopic study, we did not observe the morphological changes of cells, but the cell density decrease was observed upon post-exposure to the high concentration of CL.

## 5 Conclusion

To sum up, food additives vary in their cytotoxic and genotoxic effect. As they are typically necessary for foodstuffs, it is essential to re-evaluate. This study contributes to increasing knowledge regarding the cytotoxicity and genotoxicity effects of lactic acid salts, including CL and SL. This study showed that CL and SL could not be considered cyto-genotoxic agents at low concentrations. The cell viability assay results revealed that the proliferation of HUVECs treated with CL decreased in a time and dose-dependent manner compared to the control group. However, SL did not significantly alter the HUVECs cell number. It was indicated that CL inhibited the growth of HUVECs by inducing necrosis, verified through the FITC-labeled annexin-V assay. The result of Flow cytometry was complemented with DAPI staining and DNA ladder assays. Based upon DAPI staining and DNA ladder assays, it can be concluded that these food additives did not lead to DNA damage, shrinkage, and fragmentation in the nucleus. The central finding of the present study demonstrates that these additives can be used in foodstuff without any safety concerns. Finally, this study provides figurative and non-figurative assessments to investigate the widespread usage of food additives in the food industry.

## CRediT authorship contribution statement

**Fatemeh Javaheri Ghezel-dizaj:** Data curation, Writing – original draft, Investigation. **Maryam Ghaffari:** Software, Validation, Formal analysis. **Jafar Ezzati Nazhad Dolatabadi:** Conceptualization, Methodology, Supervision, Writing – review & editing, Visualization, Investigation. **Parvin Dehghan:** Supervision, Resources, Data curation, Project administration.

## Declaration of Competing Interest

The authors declare that they have no known competing financial interests or personal relationships that could have appeared to influence the work reported in this paper.

## Data Availability

Data will be made available on request.
